# Editorial: Capacity of the zinc mobilizing microbiome for climate-smart & sustainable agriculture

**DOI:** 10.3389/fpls.2023.1323144

**Published:** 2023-11-07

**Authors:** Izzah Shahid, Gustavo Brunetto, Felipe Klein Ricachenevsky

**Affiliations:** ^1^ Department of Biotechnology, Faculty of Science & Technology, University of Central Punjab, Lahore, Pakistan; ^2^ Department of Soil Science and Graduate Program in Soil Science, Federal University of Santa Maria, Santa Maria, Brazil; ^3^ Graduate Program in Cellular and Molecular Biology, Center of Biotechnology, Institute of Biosciences, Federal University of Rio Grande do Sul, Porto Alegre, Brazil; ^4^ Graduate Program in Botany, Botany Department, Institute of Biosciences, Federal University of Rio Grande do Sul, Porto Alegre, Brazil

**Keywords:** micronutrients, PGPR, biofortification, zinc, rhizomicrobiome

Zinc is an essential plant micronutrient needed for the regulation of physiological and metabolic processes, including oxidative metabolism, transcriptional regulation and phytohormone signaling (Ricachenevsky et al., 2015). Poor zinc availability leads to increased disease susceptibility, stunted plant growth, weak rooting system, and frail pollen formation. Use of Zn fertilizers is generally recommended to fulfil crop nutrition requirements. However, credible agronomic management practices to ensure Zn bioavailability to plants are inevitable. Besides, climate change and soil deterioration because of the persistent use of chemical fertilizers may worsen the problem. Therefore, sustainable agronomic practices are necessary to provide adequate zinc nutrition, and promote food security.

Plants can transport Zn by various transporter proteins, including ZIP family zinc-regulated transporter/iron-regulated transporter (ZRT/IRT), heavy metal ATPase family protein (HMA), vacuolar iron transporter (VIT), natural resistance-associated macrophage protein (NRAMP), and the metal tolerance protein (MTP) family (Ricachenevsky et al., 2015). These proteins are responsible for increased Zn cellular uptake, efflux, detoxification in the vacuole, and transport through other organellar membranes. In a review, Saleem et al. summarizes different strategies for enhanced Zn uptake by the plants. For example, the utilization of organic ligands can result in the formation of Zn compounds in soil leading to increased availability. Another strategy is to develop plants with modified root-traits to withstand Zn deficiency. Plants under Zn scarcity have been shown to increase lateral root development to ensure enhanced Zn uptake. Hence, plants with modified root architecture can be one of the promising strategies for improved Zn uptake and metabolism. Authors also explore the possibilities of using arbuscular mycorrhizal fungi to ensure Zn availability to plants, as well as rhizobacteria with Zn mobilization potential, and nanotechnology.

A study conducted by Ali et al. aimed at investigating the potential of indigenous bacterial strains in wheat growth promotion by increasing Zn solubilization. First, a plate-based method for identifying Zn-solubilizing bacteria was used to identify potential candidates. Selected bacterial strains showed potential as Zn solubilizers, and were tested in greenhouse experiments. Overall increase in wheat biomass was observed especially in consortium of strains, which performed better than individually inoculated ones. This study demonstrates the potential use of bacterial inoculants to promote plant growth by increasing Zn availability.

Use of synthetic fertilizers significantly modulate the response and constitution of rhizomicrobiome, alters its functioning and influence plant nutrient uptake. This hypothesis was tested by Xiao et al. in an original research paper where authors investigated the relationship between soil microbial community diversity and function, zinc concentration, and improvement in the rice yield. The data showed that application of Zn fertilizer can reduce the soil microbial communities. However, improved bacterial metabolic function lead to increased grain yield and Zn content in polished rice. Further investigation showed improvement of lipid metabolism, amino acid metabolism, carbohydrate metabolism, and xenobiotic biodegradation pathways in response to application of Zn-based fertilizers. The results showed the potential to explore interaction of rhizomicrobiome and plants following application of Zn fertilizers to yield Zn-rich grains.

Thapa et al. cataloged an extensive study using 47 biofortified and 3 non-biofortified CIMMYT-bred, bread wheat genotypes to select high-yielding genotypes with elevated grain Zn and iron concentrations. Biofortified genotypes showed high grain yield, and high Zn and iron concentration in comparison to the non-biofortified ones, with iron and Zn concentrations correlating. Heritability for grain Zn and iron concentrations was moderate to high. These results show the potential to generate biofortified, high-yield wheat genotypes that are locally adapted to farmer’s needs. It will be interesting to compare these genetic approaches with microbiome-based ones for biofortification.

Senguttuvel et al. reviewed breeding approaches for improving Zn content of rice. Plant breeders rely on selection based on additive effects, non-additive genetic effects, recovery of transgressive segregants, and hybrid generation through heterosis breeding to develop nutrient-dense biofortified rice pipelines. The authors suggested that adding target nutrition in existing and newly developed rice cultivars will be a sustainable way to address food insecurity and micronutrient malnutrition. The authors also urged that additional agronomic traits in cultivars will motivate rice breeding centers to come forward to invest in nutrition breeding. Summarizing breeding achievements, the authors highlight 29 varieties rich in Zn content as a result of global biofortification efforts. The authors also summed up the daily index of rice consumption in the countries where rice is a staple and using high Zn-based rice can lead to fulfill ~60% of the total zinc/day need.

Overall, our Research Topic includes original research articles and reviews summarizing the techniques to enhance Zn uptake in plants, innovative breeding approaches to mitigate Zn starvation, contribution of rhizomicrobiome in Zn bioavailability to plants, which will be important to determine research gaps in the field.

## Author contributions

IS: Conceptualization, Writing – original draft. GB: Writing – review & editing. FR: Writing – review & editing.
